# 3D Preoperative Planning in the ER with OsiriX^®^: When There is No Time for Neuronavigation

**DOI:** 10.3390/s130506477

**Published:** 2013-05-16

**Authors:** Mauricio Mandel, Robson Amorim, Wellingson Paiva, Marcelo Prudente, Manoel Jacobsen Teixeira, Almir Ferreira de Andrade

**Affiliations:** Department of Neurosurgery, Hospital das Clinicas of University of Sao Paulo Medical School, Av. Dr. Eneas de Carvalho, 255, São Paulo 05403-000, Brazil; E-Mails: amorim@uol.com.br (R.A.); wellingsonpaiva@yahoo.com.br (W.P.); prudente@uol.com.br (M.P.); jacobsen@usp.br (M.J.T.); alandrade@hcnet.usp.br (A.F.A.)

**Keywords:** neurosurgery, virtual surgical planning, 3-D reality, cranial traumatism, stroke, minimally invasive neurosurgery, virtual reality

## Abstract

The evaluation of patients in the emergency room department (ER) through more accurate imaging methods such as computed tomography (CT) has revolutionized their assistance in the early 80s. However, despite technical improvements seen during the last decade, surgical planning in the ER has not followed the development of image acquisition methods. The authors present their experience with DICOM image processing as a navigation method in the ER. The authors present 18 patients treated in the Emergency Department of the Hospital das Clínicas of the University of Sao Paulo. All patients were submitted to volumetric CT. We present patients with epidural hematomas, acute/subacute subdural hematomas and contusional hematomas. Using a specific program to analyze images in DICOM format (OsiriX^®^), the authors performed the appropriate surgical planning. The use of 3D surgical planning made it possible to perform procedures more accurately and less invasively, enabling better postoperative outcomes. All sorts of neurosurgical emergency pathologies can be treated appropriately with no waste of time. The three-dimensional processing of images in the preoperative evaluation is easy and possible even within the emergency care. It should be used as a tool to reduce the surgical trauma and it may dispense methods of navigation in many cases.

## 1. Introduction

The basic principle of modern neurosurgery is precise lesion localization that results in a minimally invasive approach [1–4]. To achieve this goal, various methods have been developed to define the correct position of the craniotomy and are considered standard in today's neurosurgical armamentarium [5–10]. In this sense, the use of surgical navigation systems is becoming an increasingly important part of planning and performing intracranial surgery [2,5,7]. However, there is an overvaluation of using these methods since in most cases they are only used for the craniotomy positioning [5,9]. Often, what the neurosurgeon actually need is a more accurate three-dimensional image for a proper surgical planning.

Frame-based, stereotactic craniotomy localization is the most accurate method having the lowest rate of postoperative neurological deficits and complications [8]. Although the method is the most accurate, it is time consuming, and the stereotactic frame may restrict the operating field if using microsurgical approaches. Procedures along the skull base or midline may be difficult to manage [11]. Compared with frame-based systems, frameless neuronavigation allows more flexibility during microsurgical procedures. Introduced into neurosurgical routine in the late 1980s and early 1990s, several advantages of this technology have been pointed out [7,10].

Besides the benefits of neuronavigation in tumor localization, tumor resection control, skull base surgery, or in procedures close to functional important structures, several publications pointed out that one of the most valuable applications of frameless neuronavigation was the localization of the craniotomy. Wagner and coworkers [9] showed that in 40% of the cases that had been operated on using intraoperative neuronavigation, the system was only needed to correctly define size and position of the craniotomy. This observation was confirmed in a study of Spivak and colleagues [8].

In the emergency department this reality is even more true. Frequently, due to the patients' life-threatening conditions and the need for prompt actions, there is no time for neuronavigation systems, yet, virtual reality (VR) is not time demanding and provides a much more accurate perception of the location of neurosurgical traumatic injuries [1,12,13]. VR is a computer-generated 3-dimensional (3-D) environment that provides real-time interactivity for the user [14]. The simplest form of VR is a 3-D image that can be interactively explored with a personal computer, usually by manipulating keys or the mouse so that the content of the image moves in some direction or zooms in or out. During the past decades, medical applications of VR technology have been developing rapidly, ranging from a research curiosity to a commercially and clinically important area of medical informatics and technology [4,15].

The use of the OsiriX^®^ software for advanced 3-D planning in emergency neurosurgery is described, with the aim of shedding some light on its advantages and limits [16]. The aim of this study was to show that even in an emergency situation, surgery can be customized and patient specific.

## 2. Experimental Section

In the period between January 2010 and December 2012, a total of 18 patients underwent neurosurgical interventions using the assistance of preoperative 3D planning at the Hospital das Clínicas of University of Sao Paulo Medical School. Clinical course, radiological findings and outcomes were evaluated. Neurological status was assessed using the Glasgow Coma Scale (GCS). All the patients were treated in accordance with a standard advanced trauma life support protocol if they presented directly to the emergency department of our institution.

The patient's ages ranged from 28 to 67 years (mean: 41.3 years) and there were 17 male and one female patient. Since we only present observational data of a small population size, no inferential statistics were performed.

After initial cardiorespiratory stabilization in the emergency room, computed tomography (CT) of the skull was performed immediately. If relevant Epidural Hematoma (EDH) or Subdural Hematoma (SDH) were documented the patients were brought to the operating room. The indications for surgical treatment of intracranial hematomas in our institution include hematoma volume, rapid deterioration of the level of consciousness and the presence of neurological deficits.

All CT scans were submitted to computer graphics techniques—rendering and modeling—and used to display that data as [part of] a virtual body so that it can be examined and manipulated [17]. Nowadays, commercial software/workstations that automatically complete the above-mentioned procedures are available as “low-cost” technologies [16,18–20]. Although VR technology is expensive and requires frequent updates in software and hardware, there are several low-cost options based on open-source software [16,19,20]. We use OsiriX^®^ because it is a freeware software and user friendly [16]. Additionally, it has the tools that we consider adequate for the preoperative analysis of patients described in this study.

This 3-D VR representation can be examined in detail, shared and discussed with others, and related precisely to physical reality [21]. It is up to the user to choose the amount of structures to contour, and at the end of the process, each one is already aligned to the entire volume [22–24]. Then, each object can be visualized partially or completely transparent and oriented according to the viewer's choice.

The scene is created from a range of volume data sets, surface models derived from them, and transformations derived from 3-D registrations of both volumes and models [17,25,26]. Volume rendering works directly from the available scan images, so the quality of data during acquisition will affect the result of the rendering [27].

All the available software, either commercial or open source, needs a volumetric acquisition to process the data sets [17,27–29]. In our institution, to achieve the lower coregistration errors, all volumes are acquired with the same field of view. A picture archiving and communication system (PACS) allows fast transmission of these data through a dedicated network and allows query-retrieve functions.

As we work with a group of four doctors in the emergency room (two staff neurosurgeons and two residents), surgical planning could be accomplished without prejudice on the response time for patient care. The proposal was a simple division of tasks. Thus, soon after the patient perform the CT scan, one of the doctors was responsible for copying the images using PACS and analyze with the OsiriX^®^ software. The other doctors were responsible for carrying out activities related to referring the patient to the operating room. Thus, even in the most severe cases, it was possible to perform 3D reconstruction and a rapid surgical planning.

### Planning with OsiriX

For a better understanding, we describe a step by step process of image editing:

1. Copy the files in DICOM format to a computer with the software OsiriX^®^ installed.
2. Within the software OsiriX^®^, import the DICOM files.
3. After loading all files, the next step is to create a region of interest (ROI) based on the hematoma localization, for a subsequent 3D visualization.
4. However, before this step, if the exam was not performed with a classical volumetric acquisition, it is necessary to correct the cutting angulation, otherwise the 3D model can be distorted. Click on the menu “plug-ins” and then “gantry-tilt correction”. The software automatically corrects angulation.
5. In order to create the 3D ROI simply click on the menu “ROI” and then on “growth region (2D/3D segmentation).” It is possible to create an ROI by adding the 2D images or directly forming a 3D image.
6. Now we can create the 3D model. Click on the menu “3D viewer” and then “3D volume rendering”.
7. Now we have the 3D model, but it is necessary that our ROI shows up to enable us to make correlations suitable for surgical planning. Click on the menu “ROI” and then “ROI manager”. You will see a new menu where you can select the object we have just created in the previous step.
8. Correlations with bony structures and skin structures can be made by changing the attenuation of the exam. As this is dynamic process, you can see the hematomas on transparent bone and correlate with macroscopic skin key points like the ear or the superior temporal line. The only major problem is that it is not possible to make curved-linear measurement with OsiriX. In order to decreased error we have perfomed several associative measures for the same patient (correlating with skin and bony structures) (Supplementary Material, VIDEO 1).

### Case Examples

**Figure 1.**
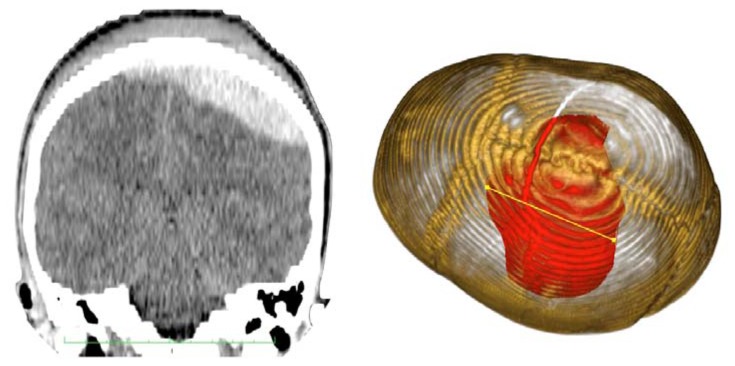
**CASE 1.** Preoperative CT scans. The visualization of the hematoma by transparency facilitates the spatial perception of the surgeon. In this particular case, the patient had an epidural hematoma beneath the superior sagittal sinus with a bilateral extension. The measured diameter is 7 cm (see also VIDEO 1).

**Figure 2.**
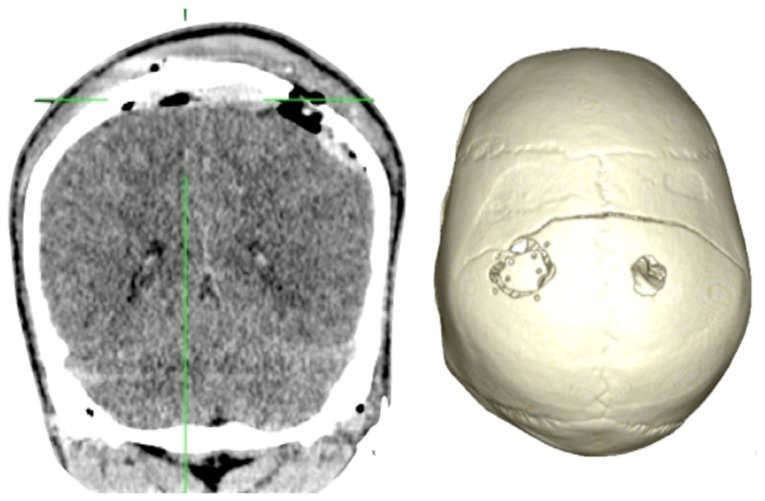
**CASE 1.** Post-operative results. We performed a combined access to this hematoma. An incision of 3 cm and a 2.5 cm craniotomy was used to resect the largest right component. An incision of 1.5 cm and a burr-hole was used to resect the left component. The component under the superior sagittal sinus was not resected to prevent possible bleeding.

Case 1-Epidural Hematoma (See Figures 1 and 2 for the details)

A 48-year-old male patient experienced traumatic brain injury due to fall from a height of 3 m. Initial loss of consciousness was noted, but the patient remained conscious and alert after the insult. He went home and was found drowsy by a family member. He was then brought to our emergency department (GCS of 14), where head CT scan revealed a bilateral parietal EDH (40 mL in volume).

The 3D reconstruction enabled a minimally invasive approach to this case. A linear incision was made at the site where the center of the hematoma is located, and a 2.5-cm diameter craniotomy was created. Another 1.5 cm incision and 1 cm burr-hole was performed in the contralateral side, leaving the hematoma beneath the superior sagittal sinus untouched. After surgery the patient had an excellent recovery with no neurological deficits. He was discharged three days after admission to the hospital.

**Figure 3.**
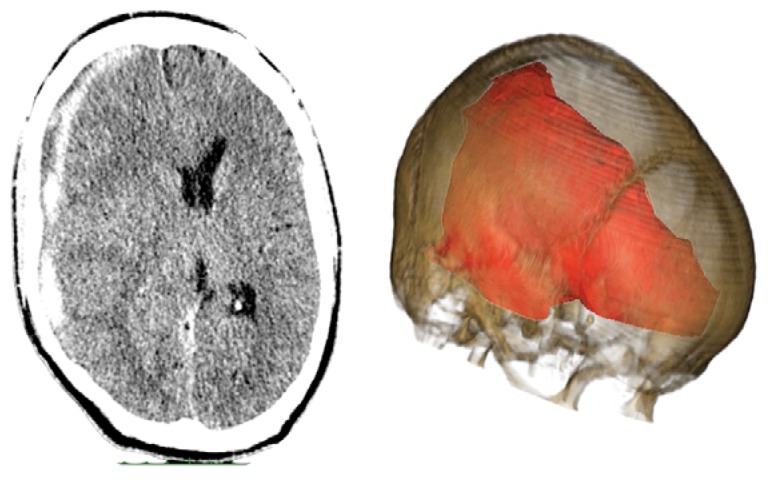
**CASE 2.** Preoperative CT scans. In patients with subdural hematomas without brain swelling is possible to perform an endoscopic resection. However the entry point is essential for a complete resection. The image makes it easier to calculate the position of the burr-hole so that the endoscope can be inserted adjacent to the hematoma over the entire brain convexity.

**Figure 4.**
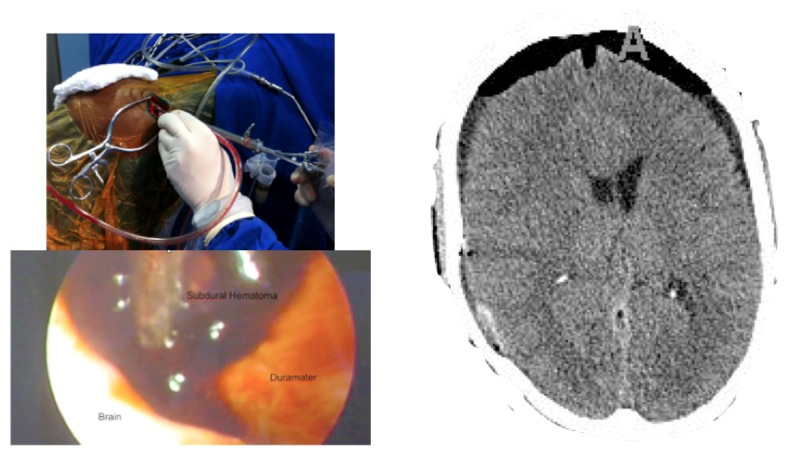
**CASE 2.** Post-operative results. During endoscopic resection is possible to insert an endoscope with aspirator or bipolar. On the left we see the positioning of the endoscope in one hand and aspirator on the other. It is also possible to see the intracranial intraoperative image. On the right, we see the postoperative CT scan with complete resection of the hematoma.

Case 2-Subdural Hematoma (See Figures 3 and 4 for the details)

A male patient of 44 years old was brought to the emergency department by relatives after falling from a height of 2 m. As the patient was well after the fall, he did not seek medical attention immediately. When he was admitted to the emergency room he presented GCS of 14 with left hemiparesis with muscle strength grade IV. The CT scan showed a subacute subdural hematoma extending over the entire convexity of the right brain. Therefore, he was promptly sent to the operating room. Upon completion of the 3D analysis it was opted for an endoscopic treatment of hematoma by a burr-hole positioned adjacent to the hematoma. The surgery was performed with no complications, and total resection was achieved. The patient had an excellent recovery, reversing the preoperative deficits. He was discharged four days after hospital admission.

**Figure 5.**
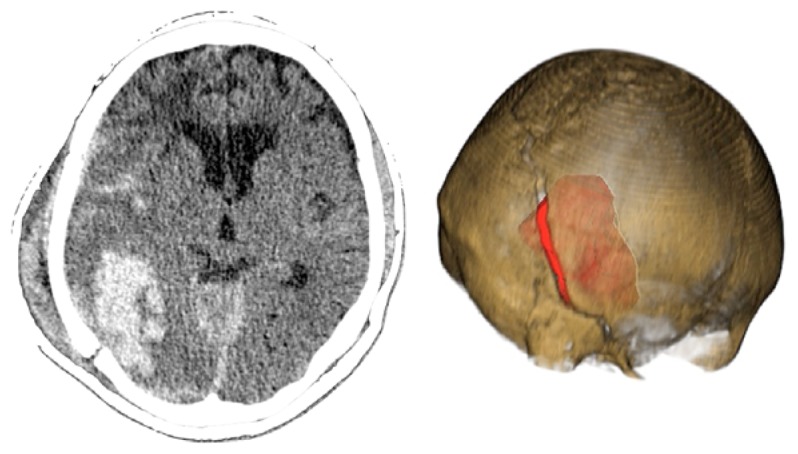
**CASE 3.** Preoperative CT scans. The 3-D environment with transparency control enables the surgeon a faster understanding of the needs and limitations of this case. Furthermore, it is possible to calculate the volume of hematoma more accurately, which helps guide the percent of hematoma was resected during surgery. In this case we prefer to avoid the site of suture disjunction.

**Figure 6.**
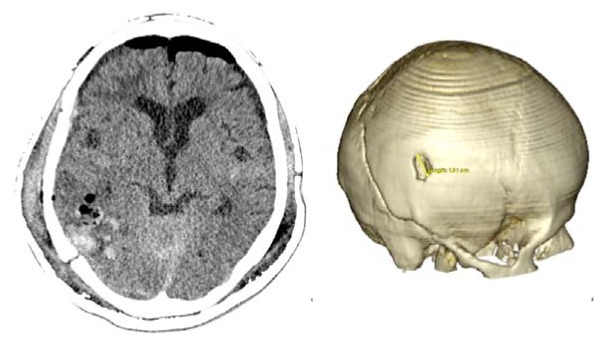
**CASE 3.** Post-operative results. The patient underwent endoscopic resection of contusional hematoma through a burr-hole. Superficial hematoma like this can be ressected without the aid of the neuroport.

Case 3-Contusional Hematomas (See Figures 5 and 6 for the details)

A 61-year-old alcoholic man was a patient who was hit by a motorcycle. Initially, he was alert and fully oriented. In the emergency department, the patient became disoriented; the examination revealed no signs of injury, and coagulation studies were normal. Within 30 min after the initial evaluation, his neurologic status deteriorated (GCS of 8). The CT scan revealed an acute right-sided post-traumatic intracranial parietal contusion containing an intraparenchymal hematoma, and acute subarachnoid hemorrhage in the right sylvian fissure. The patient underwent intubation and referred promptly to the operating room. Through the interpretation of the images 3D, we identify the best entry point for an endoscopic approach through a burr-hole. The patient had adequate recovery and was discharged seven days after surgery.

## 3. Results

Because volumetric routine CT image datasets were used, no additional CT scanning time was required for 3-D brain/cranial surface visualization. The care of patients and the time from admission to the operating room was not different from the normal average time expected in similar situations in our hospital. This occurred by division of tasks, in which a staff of neurosurgeons was responsible for surgical planning. All patients underwent minimally invasive surgical techniques based on 3D images obtained with the OsiriX^®^ software. The mean time for getting a 3D model of the patient was 10 min. We did not observe any intra or postoperative complications due to the minimally invasive approach. No patient required reoperation.

During the study period 18 patients were included. Only one patient was female. The general characteristics are summarized in Table 1. The median age was 41.3 years. The median GCS score on admission was 11. A total of 10 patients (55.5%) presented with an EDH, three (16.6%) with an SDH and five with contusional hematoma (27.7%). One patient (5.5%) presented with hematomas involving both sides, eight (44.4%) had parietal lesions, four frontal (22.2%), one exclusively temporal (5.5%), one posterior fossa (5.5%), three involving hole convexity (16.6%), two in the temporo-parietal region (11%). Most traumatic lesions were on the right site (10, 55.5%). The average volume of hematoma, measured with the OsiriX^®^ software was 35 mL.

The mechanisms of injury included falls (eight, 44.4%), bicycle accident (one, 5.5%), motorcycle accidents (four, 22.2%), car accidents (three, 16.6%), assault (one, 5.5%), and an accident involving pedestrians (5.5%). The average time between the accident and hospital admission was 92 min. This extended time reflects the fact that many patients were conscious after the accident. Therefore, they were referred to hospitals lacking neurosurgeons, and later forwarded to our hospital. The average hospital stay was 8.6 days, reflecting that most patient had less severe and isolated lesions in the brain without associated trauma to other parts of the body that could delay hospital discharge. This is also reflected by the GOS (Glasgow Outcome Scale) after 1 month of the trauma, which was mostly 5. Only three (16.6%) patients had GOS of 4 and one patient had a GOS of 3 (5.5%).

### Epidural Hematomas

We have used a minimally invasive approach for patients with epidural hematomas. The idea was to place a small craniotomy at the center of the hematoma, and the craniotomy diameter was half the overall diameter of the hematoma. Thus, it was possible to make only small straight incisions (mean 3.5 cm). The average diameter of hematomas was 6 cm and the mean of the volumes of hematomas was 31.5 mL. The vast majority of patients had a favorable outcome, as is to be expected in patients with uncomplicated epidural hematomas.

### Subdural Hematomas

Only three patients underwent surgery with the diagnosis of subdural hematoma, both acute (two patients), and subacute (one patient). All patients had lesions that extended all over the brain convexity with volumes ranging from 27 to 40 mL. All patients underwent an endoscopic resection through a burr-hole of 2 cm in diameter. Through the burr-hole, the neuroendoscope and another instrument (bipolar or aspirator) were introduced. The location of the bur-hole was calculated using the 3D model made immediately before surgery. The idea was place it at the periphery of the hematoma so that the aspirator and the endoscope could be inserted adjacent to the hematoma. This strategy allowed the complete resection of all hematomas without complications. All patients had a good outcome in view of the gravity that represents a subdural hematoma.

### Contusional Hematomas

Contusional hematomas were operated with endoscopic technique already described by several authors for spontaneous intraparenchymal hematoma [3,30–32]. The observation of 3D images enabled us to choose the best location (far from eloquent areas and with less brain tissue between the hematoma and the brain surface), to insert the “neuroport”. The neuroport is a canula used to dissect the brain tissue till the hematoma causing less injury to the adjacent brain tissue [3,30–32].

The resection of the hematoma followed the same line of the spontaneous hematomas [3,30–32]. All hematoma were resected satisfactorily. However, despite achieving adequate decompression, three patients had residual hematoma on the control CT scan. None required reoperation. There was no difference or specific difficulty. As seen in Table 1, only one patient, who had also a contralateral acute subdural hematoma had a bad outcome (GOS of 3).

## 4. Discussion

One of the many skills that must be developed by brain surgeons is the spatial reconstruction and integration of 2-D pictures within the anatomic reality. Surgeons can view these images using specific 2-D image viewers. Using a number of these image slices, surgeons build their own mental 3-D model of the anatomy and pathology (*i.e.*, a tumor and its neurovascular relationships) [33,34]. Furthermore, the surgeon should be able to reconstruct in his/her mind how different information originating from different imaging modalities is spatially interconnected [11,17,33]. This task can be difficult, even for experienced surgeons, because pathological processes can contribute to modifying and increasing interindividual anatomic variability, thus further complicating the task. As a consequence, they can miss important information or draw incorrect conclusions that can lead to suboptimal treatment strategy decisions.

The current standard of care for lesion localization during surgical planning and microsurgical dissection is based on 2-D CT and MR images and navigation devices. These 2-D images enable the surgeon to use past experience to mentally construct a surgical approach to the lesion and predict the 3-D variability that may be encountered during surgery. It has been well documented that surgical outcome, in part, depends on the number of cases, and hence experience [33]. In this sense, the neurosurgery of patients in the emergency department occurs in a standardized way so that time is optimized and the surgeon does not have to consider nuances of planning. Currently this is the standard of care. However, our results point to an interesting alternative.

There is a possibility accomplish specific patient surgeries accurately. The use of three-dimensional reconstruction to maximize the understanding of the pathology has increased the accuracy of the surgeon. Moreover, although it seems paradoxical, it actually shortens the interpretation of imaging studies [35]. “A picture is worth a thousand words”. We demonstrated in our cases that the average time for the three-dimensional planning was 10 min and there was no prejudice to the time of patient care, especially when we consider a division of tasks in the neurosurgical team. Thus, even less experienced neurosurgeons can draw conclusions and apply them to the surgical planing quicker. The only disadvantage of OsiriX^®^ software is its inability to make curved-linear measurements. In order to reduce our error, we have perfomed several associative measures for the same patient. We have utilized bony structures like the zygoma, superior temporal line, cranial sutures and skin structures like the ear, eyebrown and the nose. As the measures do not need to have a millimetric precision, these actions were sufficient to get the correct mini-craniotomy location in relation to the hematomas.

The possibility that we may have an X-ray vision, enabling the neurosurgeon to see and interact with the precise location of the hematoma through a 3D model of the skull, represents a breakthrough that cannot be overlooked or underutilized [36–39]. Furthermore, the incorporation of this technology allows a precision that enables the performance of minimally invasive procedures with similar success rates to established procedures [1,4]. Though still we need controlled studies to demonstrate the true efficacy of these concepts, some advances can already be considered safe in the emergency department.

The results provide evidence that 3-D visualization of the cranial surface is superior to 2-D for brain trauma surgery. The proposed method for 3-D brain surface visualization does not require in-depth computational knowledge and has acceptable preparation time [40]. The 3-D visualization was accurate and fast. The resulting 3-D images were shown to be useful for intra-operative orientation to the exact position of the craniotomy independent from patient urgency.

The use of 3-D visualization based on segmentation of strategic structures can improve surgeons' understanding of the complex pathological relationships between vital structures and surgical targets. Since the first reports [41], advances in computer processing capabilities and hardware manufacturing have led to widespread use of VR and its implementations such as augmented-reality systems [42–46].

The presented data suggest that 3-D reconstruction of the CT images can aid the neurosurgeon in precise and fast topographic lesion localization for surgical planning and intraoperative orientation. Unlike other authors, we have used OsiriX^®^ (open-source software) with direct volume rendering capability, because it is broad available and inexpensive [16]. Direct volume rendering is a method for visualization of a stack of medical images. Direct volume rendering can greatly facilitate planning an intervention, because the neurosurgeon can perform a patient-specific virtual approach before the actual operation. The 3-D model can be moved, rotated, and zoomed into a position that correlates with the intraoperative positioning of the patient's head and brain. The clot removal and/or corticotomy can be simulated by using a slicing function [11,17,28,39].

Additionally, it is interesting to note that there are nuances between the different traumatological entities. We have described the application in epidural hematomas, subdural hematomas and contusional hematomas. It is necessary to emphasize the importance of three-dimensional reconstruction in each case. In epidural hematomas, this type of tool allowed us to accurately position the craniotomy in the center of the hematoma, reducing its size without prejudice of the resection. In Subdural hematomas, three-dimensional images were essential to position the burr-hole on the periphery of the hematoma so that the endoscope can be inserted adjacent to the hematoma. In contusional hematomas, the optimal positioning of the burr-hole is facilitated by 3D images.

This method demonstrated here does not need the effort of setting up an image-guided navigation system. It simply relies on the transfer of a surgical plan carried out on a virtual patient model onto the actual head of the patient in the operating room. The prerequisites for such a procedure are accurate 3D planning tools including the graphical reconstruction of a precise skin surface and curved-linear measurement tools that follow the skin's curvature.

We have to remember that neuronavigation is not only used for craniotomy placement but also assists in intra-operative orientation, tumor localization, resection control, or helps in finding the optimal trajectory for endoscopic approaches [2,5,7,9]. These aspects are not covered by VR-aided surgery planning. However, this method of meticulous surgery planning might serve as a backup enabling the surgeon to correctly place the craniotomy for a minimally invasive procedure in a trauma set, where there is no time for placing a neuronavigation system.

## 5. Conclusions/Outlook

Three-dimensional visualization of cranial surface anatomy, when added to standard patient care, is extremely useful for intraoperative orientation to the exact position of the craniotomy, independent from the traumatic pathology. Advanced 3-D planning provides useful information that can facilitate the learning curve of spatial orientation in neurosurgery. Based on the present study, 3-D cranial surface visualization can be considered as an additional reliable tool for preoperative lesion localization, patient-specific trajectory planning, and intraoperative guidance for brain trauma surgery. Its use covers all the main fields of emergency neurosurgery and contributes to the development of a sort of surgery that is more patient-specific and minimally invasive.

## Figures and Tables

**Table 1.** Results. Legends: A = Age; ΔT (admission) = time until the patient was admitted to the hospital; LOC = location of the traumatic lesion (F = frontal; P = parietal; T = temporal; FTP = fronto-temporo-parietal; TP = temporo-parietal; PF = posterior fossa); LAT = lateralization of the traumatic lesion (R = right; L = left; B bilateral); V = traumatic lesion volume; ΔD hematoma = diameter of the hematoma (only for epidural hematomas); ΔD incision = incision length; ΔD craniotomy = diameter of the craniotomy; ΔT neuro = time of hospital stay due to neurological status; ΔT hospital = hole hospital stay time; GOS = Glasgow outcome scale after 1 month of the accident; TSAH = Traumatic Subdural Acute Hemorrhage; C Fracture = Cranial Fracture.

**Patient**	**A**	**GCS**	**ΔT (admission)**	**LOC**		**LAT**	**V (mL)**	**Associated Lesions**	**ΔD Hematoma**	**ΔD Incision**		**ΔD Craniotomy**	**ΔT Hospital Stay**		**GOS**
**Epidural Hematomas**															
PJ	44	15	4 h	P		R	26	TSAH	6 cm	3 cm		2.8 cm	4 days		5
GM	48	14	3 h	P		B	40	TSAH	7 cm	3 cm		2.5 cm	3 days		5
MG	28	14	2 h	TP		R	34	---	8 cm	3 cm		2.8 cm	4 days		5
ES	32	15	1 h	P		R	25	TSAH	5 cm	3 cm		2.0 cm	3 day		5
ARB	28	13	1h	P		R	34	---	5 cm	4 cm		3 cm	3 days		5
EV	30	14	40 min	F		L	33	---	5.5 cm	5 cm		3.0 cm	5 days		5
GA	41	14	30 min	TP		L	40	---	6.7 cm	4 cm		3.5 cm	4 days		5
LS	32	6	1 h	P		L	20	F. Epidural	4 cm	3 cm		2.5 cm	5 days		5
ISLA	45	6	30 min	F		L	25	Right Subdural	5 cm	4 cm		2 cm	14 days		5
FN	56	13	30 min	P		L	38	Parietal Contusion	7 cm	3 cm		2.5 cm	20 days		4
**Acute And Subacute Subdural Hematoma**															
AC	44	10	1 h	FTP	R		27	TSAH	---	3 cm		2 cm	4 day		5
EN	39	6	30 min	FTP	R		35	---	---	3 cm		2 cm	5 days		4
AC	55	14	2 days	FTP	R		40	---	---	3 cm		2 cm	5 days		5
**Contusional Hematoma**															
GM	44	14	4 h	F	L		35 mL	TSAH	---		3	1.5 cm		5 days	5
MCS	67	8	3 h	P	R		25 mL	TSAH	---		3	2 cm		15 days	5
BB	61	8	2h	P	R		42 mL	TSAH/P. Fracture	---		3	2.8 cm		7 days	4
CP	32	12	1h	T	L		44 mL	C. Fracture	---		4	1.5 cm		9 day	5
LAP	28	6	1h	F	R		34 mL	Subdural Hematoma	---		5	1.5 cm		21 days	3
